# Psychometric properties of self-sufficiency assessment tools in adolescents in vocational education

**DOI:** 10.1186/s40359-015-0091-2

**Published:** 2015-09-25

**Authors:** Rienke Bannink, Suzanne Broeren, Jurriën Heydelberg, Els van’t Klooster, Hein Raat

**Affiliations:** Department of Public Health, Erasmus University Medical Center Rotterdam, P.O. Box 2040, 3000 CA Rotterdam, The Netherlands; Municipality of Rotterdam, Librijesteeg 4, 3000 KS Rotterdam, The Netherlands; Public Health Care for Youth, Westblaak 171, 3012 KJ Rotterdam, The Netherlands

**Keywords:** Psychometrics, Reliability, Validity, Self-sufficiency, Adolescents, Self-sufficiency matrix

## Abstract

**Background:**

Self-sufficiency is the realisation of an acceptable level of functioning either by the person him/herself or through the adequate organisation of help from informal or formal care providers. Assessment of self-sufficiency for determining an individual’s functional strengths and areas for improvement is increasingly being applied among adolescents in vocational education, a group considered vulnerable with high school dropout rates and often characterised by an accumulation of problems. This study examined the psychometric properties of two instruments, i.e. a self-report questionnaire assessing self-sufficiency and the Self-Sufficiency Matrix for professionals (SSM-D) conducted among adolescents in vocational education.

**Methods:**

The self-report questionnaire used to assess self-sufficiency was completed by 581 adolescents. Professionals completed the SSM-D for 224 of the 581 adolescents. Furthermore, constructs related to the domains of self-sufficiency were assessed with self-report questionnaires and information about school absenteeism was monitored via the school registration system.

**Results:**

For both self-report and professional-report ratings, the internal consistency was satisfactory (Cronbach’α > 0.70) and various minor to strong correlations were found between the domains of self-sufficiency and related constructs. For most of the domains, there was little or no agreement between professionals and adolescents.

**Conclusions:**

Both the self-report questionnaire assessing self-sufficiency and the SSM-D applied in this study seem to possess adequate psychometric properties. The results indicated that adolescents and professionals provide different views of adolescents’ self-sufficiency, which merits further study. In the meantime, we recommend assessment of adolescents’ self-sufficiency by using both the self-report questionnaire and the SSM-D to get a comprehensive measure of adolescents’ self-sufficiency.

**Trial registration:**

Netherlands Trial Register: NTR3545; 30 July 2012.

**Electronic supplementary material:**

The online version of this article (doi:10.1186/s40359-015-0091-2) contains supplementary material, which is available to authorized users.

## Background

Mental health problems are highly prevalent in adolescents, and risk behaviours, such as substance abuse and truancy, are often acquired during adolescence [1]. These problems and behaviours can negatively affect the functioning of adolescents in different life domains [2]. Furthermore, mental health problems and risk behaviours often do not occur in isolation in adolescents, but are associated with each other and accumulate [3–9]. The co-occurrence of mental health problems and risk behaviours, and the influence that these problems and behaviours have on the functioning of adolescents in various life domains, suggests that professionals should preferably address problems and risk behaviours in multiple life domains simultaneously. However, to date most intervention programmes and assessment tools take a single-problem/risk-behaviour/life-domain approach instead of an integrated approach [10].

A Self-Sufficiency Matrix (SSM) is an instrument that has adopted such an integrated approach [11, 12]. The basis for the SSM was developed in the 1990s in the United States. It is a standardised tool for measuring self-sufficiency. Self-sufficiency is defined as the realisation of an acceptable level of functioning either by the person him/herself or through the adequate organisation of help from informal or formal care providers [2]. A standardised tool to measure economic self-sufficiency was first developed by Pearce et al. [13]. This economic self-sufficiency measure was then extended to include a number of domains, resulting in the first published version of a multidimensional SSM in 2004 [14]. Different versions of the SSM are currently being used in different settings. The SSM can be used by professionals as a screening tool during consultations for determining functional strengths and areas for improvement in, for example, vulnerable adolescents. It expresses functioning in terms of levels of self-sufficiency in several domains (e.g. mental health and social network) [2]. The SSM is a screening or assessment tool that is often used also to measure outcomes of intervention programmes in populations experiencing multiple interlinked problems.

Although the SSM is applied in the United States [11, 12] and is quickly gaining popularity in other countries as well [15], to the best of our knowledge there is only one study available that examines the psychometric properties of the SSM. Fassaert et al. [2] showed that an adapted 11-domain version of the SSM (SSM-D), based on Utah and Arizona versions of the SSM, is a reliable instrument for assessment by professionals of the self-sufficiency of adolescents (>18 years) with severe and complex psychiatric problems. As the SSM is also increasingly used among other populations, such as adolescents in vocational education (≥15 years), further evaluation of the psychometric properties of the SSM among other populations is needed. This study focuses on these adolescents in senior vocational education, a group that is considered vulnerable. In the Netherlands, 75 % of school dropouts occur in senior vocational education [16]. Furthermore, many adolescents in vocational education experience problems, such as debts and substance abuse, and these problems often accumulate [3, 4, 17].

So far, the SSM is only available for professionals to complete during consultations. However, previous research has shown low correlations between different informants (e.g. adolescents and professionals) when assessing problems, and that a valuable unique contribution can be made by different informants [18–22]. Hence, assessment of self-sufficiency by means of a questionnaire for adolescents alongside a proxy rating by a professional could give a more comprehensive measure of adolescents’ self-sufficiency. Therefore, this study employed assessment of self-sufficiency by means of a questionnaire for adolescents in addition to assessment of self-sufficiency by a proxy rating provided by professionals.

The purpose of this study was to assess the psychometric properties of a self-report questionnaire assessing self-sufficiency and the SSM-D in a group of vulnerable adolescents (i.e. in vocational education). This study investigated: (1) internal consistency of both instruments assessing self-sufficiency (i.e. self-report questionnaire and SSM-D), and (2) correlations between adolescents’ and professionals’ ratings in domains of self-sufficiency and related constructs (concurrent validity). Additionally, we examined the degree of agreement between adolescent and professional ratings in the domains of self-sufficiency.

Since there are some conceptual differences between the domains of self-sufficiency and the related constructs that were used to assess concurrent validity (e.g. finances and debts), minor to strong correlations are expected depending on the level of overlap between the constructs under study. In line with previous studies on adolescents’ psychopathology that measured agreement between informants [18, 19, 21, 22], we hypothesise that the degree of agreement between adolescents and professionals in the domains of self-sufficiency will be fair at most. Low levels of agreement between adolescents and professionals could indicate that these informants cannot be substituted for one another because they provide unique information [18].

## Methods

### Data collection

This study used data obtained from enrolments in the Your Health study, a cluster randomised controlled trial (Trial registration: www.trialregister.nl; Netherlands Trial Register: NTR 3545; 30 July 2012). A total of 44 first-year classes of students in vocational education in the Rotterdam region of the Netherlands participated. School classes (clusters) were randomly assigned to the Your Health or the control condition. The intervention study itself is described in detail elsewhere [23]. A few weeks prior to the start of the study, all adolescents and parents received information about the study. Parents were asked passive written informed consent. If parents did not want their child to participate, and their child was not yet 18 years old, they could object to the child’s participation. During a classroom session, adolescents who were present in class were asked to provide active written informed consent before they completed a set of questionnaires. The set of questionnaires included the self-report questionnaire assessing self-sufficiency and questionnaires assessing the related constructs. After the questionnaires had been administered, school classes were randomly assigned to the Your Health or the control condition. Adolescents in the intervention group were invited to attend a preventive health consultation with the school nurse. During this consultation, the nurse used the SSM-D and rated the self-sufficiency of the adolescent.

**Fig. 1:**
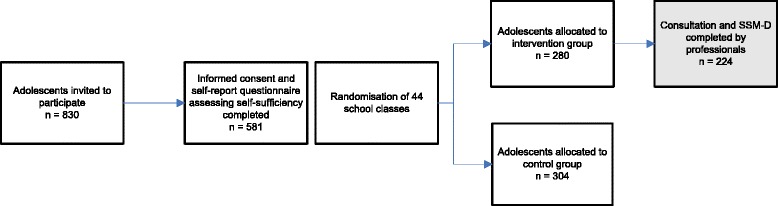
Flow chart of the adolescent’s participation

Of the 830 adolescents who received information about the study, 584 (70.4 %) were present at the time of assessment, provided written informed consent and participated; 280 in the Your Health group and 304 in the control group. The main reason for non-participation was absence at the time of the assessment. The questionnaire used to assess self-sufficiency was completed by 581 of the 584 (99.5 %) participating adolescents. Of the 280 adolescents who were invited to attend a consultation, 224 (80.0 %) attended (see Fig. 1).

## Ethics statement

The Medical Ethical Committee of Erasmus MC has reviewed the research proposal for this study and declared that this study does not fall within the ambit of the Medical Research Involving Human Subjects Act (also known by its Dutch abbreviation “WMO”) and, therefore, does not require further approval of an ethics review board. The Medical Ethical Committee had no objection against the execution of this research proposal (MEC-2012-367).

### Measurements

#### Assessment of self-sufficiency by professionals

The Dutch version of the SSM (SSM-D) was used to assess an individual’s level of self-sufficiency in 11 life domains: finances, day-time activities, housing, domestic relations, mental health, physical health, addiction, activities daily life, social network, community participation, and judicial [24, 25]. Each of the domains was measured by a single item and the level of self-sufficiency was rated on a 5-point scale: 1 = ‘*acute problem*’, 2 = ‘*not self-sufficient*’, 3 = ‘*barely self-sufficient*’, 4 = ‘*adequately self-sufficient’,* and 5 = ‘*completely self-sufficient*’. Indicators that specify each level of self-sufficiency were defined for each domain. Together, these indicators form a matrix of domains and levels of self-sufficiency [2, 24]. For an example of the indicators of an SSM-D domain (i.e. finances), see Table 1. Prior to the consultations, nurses were trained to work with the SSM-D.

**Table 1:** Example of an indicator in the Dutch Self-Sufficiency Matrix: Finances

Rating	Label	SSM-D description
1	Acute problem	No income. High, increasing debts.
2	Not self-sufficient	Insufficient income and/or spontaneous or inappropriate spending. Increasing debts.
3	Barely self-sufficient	Can meet basic needs with income and/or appropriate spending. If there are debts, they are at least stable and/or controlled by a third party.
4	Adequately self-sufficient	Meets basic needs without receiving social security benefits. Manages possible debts without assistance and they are decreasing.
5	Completely self-sufficient	Income is ample, well managed. Has the ability to save with income.

Note: Copyright 2012 by GGD Amsterdam. Reprinted with permission

#### Assessment of self-sufficiency by adolescents

A self-report questionnaire assessing self-sufficiency was developed based on the 11-domain version of the SSM-D. Each domain name was translated into simple language, and a short description was provided describing the content of each domain in simple language. Simple language was used because some adolescents may have relatively poor reading skills. Subsequently, based on group discussions and consensus between professionals, language adjustments were made and the response scale of the professional version was simplified. Professionals indicated that the word ‘self-sufficiency’, used in the response scale of the SSM-D, would be too difficult for adolescents to understand. Therefore, the response scale was replaced in a simple 5-point Likert scale: 1 = ‘*no problems’*, 2 = ‘*few problems*’, 3 = ‘*not few/not many problems’*, 4 = ‘*many problems’*, and 5 = ‘*very many problems’*. Furthermore, a smiley was displayed with each response option to support adolescents with poor reading skills. Finally, a pilot was conducted among the target group (i.e. adolescents in vocational education) to examine whether the language and the response scale used were clear, and whether the instrument was usable in this group. No further adjustments were needed based on this pilot.

Our self-report questionnaire differs in some respects from the SSM-D. First, the self-report questionnaire provides a short description for each domain, but it does not define any indicators specifying each level of self-sufficiency as is the case in the SSM-D. Second, the self-report questionnaire has a different 5-point response scale (with smileys) than the SSM-D. For an example of a domain of the self-report questionnaire (i.e. finances), see Table 2.

**Table 2:** Example of a self-sufficiency domain in the self-report questionnaire: Finances

Description	Rating				
Did you experience problems getting by financially over the past six months?	No problems ☺ ☺	Few problems ☺	Not few/not many problems ☹	Many problems ☹	Very many problems ☹☹

#### Demographics

Demographic characteristics included the age, gender, country of birth of the adolescent and both parents, and whether or not the adolescent already was a parent him/herself. Ethnicity was classified as Dutch or non-Dutch, in accordance with the definitions used by Statistics Netherlands [26].

#### Related constructs

Debts, homelessness, alcohol consumption, soft drug use and delinquency were assessed by items based on existing instruments previously developed by Municipal Public Health Services and health institutes in the Netherlands [27]. To reduce respondent burden, only a number of related construct were assessed. No data was obtained on 3 domains of self-sufficiency (i.e. domestic relations, activities daily life, and social network).

#### Debts

Debts were assessed on an ordinal scale by the following items: (1) do you have debts? (yes/no/don’t know), and (2) approximately how high is the sum of all your debts? (less than 50 euros – more than 2,500 euros).

#### Homelessness

Homelessness was assessed by the item: “Have you been homeless in the past three months? This means that you had no perspective, for at least one night per month, of a permanent place to sleep.” (yes/no).

#### Alcohol and soft drugs

Alcohol consumption was covered by the following two items: (1) how often have you drunk five or more alcoholic drinks on a single occasion over the past four weeks? (never – nine or more times), and (2) how often have you been drunk or tipsy over the last four weeks? (never – 20 or more times). Soft drug use was assessed by how often the adolescent had used soft drugs over the previous four weeks (never – 20 or more times).

#### Delinquency

Delinquency was assessed by the item: “In the past 12 months, have you been questioned at a police station because you were accused of doing something that was not permitted?” (never – 6 or more times).

#### Mental health status

Mental health status was assessed by the Mental Health Inventory (MHI-5) [28]. The MHI-5 includes five questions referring to both positive and negative aspects of mental health. All questions contain six possible response categories, scored between 1 and 6. The total score is transformed into a variable range of 0–100, with a score of 100 representing optimal mental health (current study α = 0.69).

#### Depressive symptoms

Symptoms of depression were assessed by the Center for Epidemiologic Studies Depression Scale (CES-D) [29]. The CES-D consists of 20 items. The frequency of symptoms is rated on a 4-point scale ranging from 0–3. Items scores are summed (range from 0–60), with higher scores indicating higher levels of depressive symptoms (current study α = 0.89).

#### Health-related quality of life

Health-related quality of life was assessed by the Short Form-12 Health Survey (SF-12). The SF-12 consists of 12 items, with variable response categories across the items. The scores are summarised into two components, corresponding to mental and physical health-related quality of life, with scores ranging from 0 (worst possible health state) to 100 (best possible health state) (current study α = 0.72).

#### School absenteeism

In the school registration system every hour of absence was registered either as permitted (i.e. because of illness or another valid reason) or not permitted (i.e. without notification or valid reason). Absenteeism was defined as the number of hours adolescents were absent (permitted or not permitted) in a 2-month period around the administration of the questionnaire.

### Statistical analyses

Internal consistency was assessed by Cronbach’s alpha, for which a value of ≥0.70 was considered adequate [30]. To determine concurrent validity, ratings for eight domains of both instruments (i.e. self-report questionnaire and SSM-D) assessing self-sufficiency were compared to ratings for related constructs. Concurrent validity was assessed by calculating the rank biserial, polychoric, or polyserial correlation between each domain and related constructs. Rank biserial correlation (r_rb_) is used to determine the correlation between an ordinal and dichotomous variable. Polychoric correlation (r_pc_) is used to determine the correlation between two ordinal variables, and polyserial correlation (r_ps_) is used to determine the correlation between a continuous and an ordinal variable [31]. Furthermore, concurrent validity was assessed by calculating Pearson correlations (r) between the total score on SSM-D (which ranges from 11–55) and related constructs, and between the total score on the self-report questionnaire (which ranges from 11–55) and related constructs. The criteria for judging the size of the correlation coefficient suggested by Cohen were applied: correlations <0.30 are considered minor, correlations between 0.3 – 0.49 are considered medium, and ≥0.5 are considered strong [32].

The degree of agreement between professionals and adolescents in each of the domains was determined with weighted kappa with linear weights. Weighted kappa is a measurement of agreement for categorical data with an ordinal level [33]. Linear weighting is used when the difference between each category has the same importance. According to Altman’s guidelines [34], *K* is poor when it has a value of ≤0.20, fair when it is between 0.21–0.40, moderate when it is between 0.41–0.60, and good when it is ≥0.60.

Statistical analyses were performed using SPSS version 21. Polyserial, polychoric, and rank biserial correlations were calculated in SAS version 9.3. Additionally, polyserial correlations between each domain of self-sufficiency and the total score on SSM-D, and between each domain and the total score on the self-report questionnaire, were assessed (see Addtional file 1).

## Results

### Adolescents’ characteristics

The self-report questionnaire assessing self-sufficiency was completed by 581 adolescents. The average age of these adolescents was 18.3 years (SD = 2.60); 39.0 % were male, 28.1 % were of Dutch ethnicity, and 10.6 % were parents (Table 3). Professionals completed the SSM-D for 224 of these adolescents. The average age of this subsample of adolescents was 18.3 years (SD = 3.59); 41.7 % were male, 25.3 % were of Dutch ethnicity, and 12.2 % were parents.

**Table 3:** Demographic characteristics of the study population

	Self-sufficiency		
	Completed questionnaires - Adolescents		Completed SSM-D - Professionals
	Total group	Intervention group	
Number (n)	581	280	224
Mean age; years (SD)	18.27 (2.60)	18.46 (2.65)	18.26 (2.59)
Gender of adolescent (male, %)	39.0	43.0	41.7
Ethnicity (Dutch, %)	28.1	24.9	25.3
Being a parent (yes, %)	10.6	13.4	12.2

### Self-sufficiency

A score of “not to barely self-sufficient” can be seen as a level of self-sufficiency that can be improved. The domains in which the professionals deemed the highest percentages of adolescents as being “not to barely self-sufficient” were community participation (36.7 %), domestic relations (15.8 %) and social network (14.5 %) (Table 4). The domains in which the adolescents themselves deemed the highest percentage of adolescents as being “not to barely self-sufficient” were different, namely, finances (23.3 %), domestic relations (17.4 %) and mental health (16.7 %).

**Table 4:** Professionals’ and adolescents’ ratings of self-sufficiency (*n* = 224)

	Not to barely self-sufficient^a^	Acute problem	Not self-sufficient	Barely self-sufficient	Adequately self-sufficient	Completely self-sufficient
	%	%	%	%	%	%
*Professionals’ ratings (n = 224)*	1–3	1	2	3	4	5
Finances	12.9	0.9	4.9	7.1	42.4	44.6
Day-time activities (*n* = 1 missing)	1.3	0.0	0.0	1.3	85.2	13.5
Housing (*n* = 3 missing)	9.0	0.0	1.4	7.7	26.7	64.3
Domestic relations (*n* = 2 missing)	15.8	0.5	3.2	12.2	25.7	58.6
Mental health (*n* = 1 missing)	8.5	0.0	0.4	8.1	22.4	69.1
Physical health	4.5	0.0	0.4	4.0	29.0	66.5
Addiction	3.6	0.0	0.0	3.6	34.8	61.6
Activities daily life (*n* = 2 missing)	4.5	0.0	0.0	4.5	29.3	66.2
Social network (*n* = 3 missing)	14.5	0.9	1.8	11.8	43.4	42.1
Community participation (*n* = 3 missing)	36.7	0.5	12.7	23.5	47.1	16.3
Judicial (*n* = 1 missing)	12.6	0.0	5.4	7.2	18.4	69.1
	Not to barely self-sufficient^a^	Very many problems	Many problems	Not few/ not many problems	Few problems	No problems
	%	%	%	%	%	%
*Adolescents’ rating (n = 224* ^*b*^ *)*	1–3	1	2	3	4	5
Finances (*n* = 1 missing)	23.3	4.9	5.4	13.0	23.3	53.4
Day-time activities (*n* = 2 missing)	8.1	0.5	1.4	6.3	19.8	72.1
Housing (*n* = 1 missing)	12.1	2.2	2.2	7.6	6.3	81.6
Domestic relations	17.4	2.7	4.0	10.7	17.9	64.7
Mental health (*n* = 3 missing)	16.7	3.2	4.1	9.5	17.2	66.1
Physical health (*n* = 2 missing)	11.3	1.8	2.3	7.2	18.9	69.8
Addiction (*n* = 5 missing)	7.8	0.9	1.4	5.5	9.1	83.1
Activities daily life	6.3	0.4	0.9	4.9	10.3	83.5
Social network (*n* = 1 missing)	8.1	0.9	0.9	6.3	12.6	79.4
Community participation (*n* = 3 missing)	8.1	0.9	2.3	5.0	14.9	76.9
Judicial	5.4	0.4	0.9	4.0	7.1	87.5

^a^A rating of ≤ 3 is considered as not to barely self-sufficient

^b^In this table, only ratings for adolescents for whom a professional rating was available are displayed (*n* = 224)

### Internal consistency

Internal consistency was adequate. The Cronbach’s alpha of the self-report questionnaire was 0.84 and of the SSM-D 0.71.

### Concurrent validity

Various minor to strong correlations were found between domains and related constructs (Table 5). All significant correlations were in the hypothesised direction. Correlations between professionals’ ratings of self-sufficiency in the different domains and related constructs varied from no correlation to strong correlations. The strongest correlations were found between the domains of finances and debts (r_pc_ = −0.66), the domains addiction and soft drug use (r_pc_ = −0.53), the domains addiction and alcohol consumption (drunk or tipsy) (r_pc_ = −0.41), and the domains judicial and delinquency (r_pc=_-0.41).

**Table 5:** Concurrent validity: correlations between professionals’ and adolescents’ ratings of self-sufficiency and related constructs

Self-sufficiency	Related constructs	Correlation	
		With professionals’ self-sufficiency rating (*n* = 224)	With adolescents’ self-sufficiency rating (*n* = 581)
Total self-sufficiency score	Mental health-related quality of life (SF-12)^a^	0.21^b^	0.46^b^
	Physical health-related quality of life (SF-12)^a^	0.12^b,f^	0.28^b^
Finances	Debts	−0.66^c^	−0.74^c^
Day-time activities	Not-permitted school absenteeism	−0.26^d^	−0.17^d^
	Permitted school absenteeism	0.01^d,f^	−0.11^d^
Housing	Homelessness	−0.41^e,f^	−0.39^e^
Mental health	Mental health status (MHI-5)^g^	0.30^d^	0.60^d^
	Depressive symptoms (CES-D)	−0.33^c^	−0.59^d^
	Mental health-related quality of life (SF-12)^a^	0.29^d^	0.54^d^
Physical health	Physical health-related quality of life (SF-12)^a^	0.10^d,f^	0.33^d^
	Permitted school absenteeism	−0.08^d,f^	−0.13^d^
Addiction	Alcoholic drinks: 5 or more on 1 occasion	−0.30^c^	−0.39^c^
	Alcohol: drunk or tipsy	−0.41^c^	−0.53^c^
	Soft drug use	−0.53^c^	−0.53^c^
Community participation	Not-permitted school absenteeism	−0.20^d^	0.03^d,f^
	Permitted school absenteeism	−0.11^d,f^	−0.04^d,f^
Judicial	Delinquency	−0.41^c^	−0.58^c^

^a^A higher score indicates a better quality of life

^b^Pearson correlation

^c^Polychoric correlation

^d^Polyserial correlation

^e^Rank biseral correlation

^f^Non-significant correlations; all other correlations were significant at *p* <0.05

^g^A higher score indicates less mental health problems

Correlations between adolescents’ ratings of self-sufficiency in the different domains and related constructs also varied from no to strong correlations. Comparable with correlations between professionals’ ratings and related constructs, the strongest correlations between adolescents’ ratings and related constructs were found between the domains of finances and debts (r_pc_ = −0.74), the domains addiction and soft drug use (r_pc_ = −0.53), the domains addiction and alcohol consumption (drunk or tipsy) (r_p_ = −0.53), and the domains judicial and delinquency (r_pc=_-0.53). In addition, strong correlations were found between the domains of mental health and mental health status (r_ps_ = 0.60), depressive symptoms (r_ps_ = −0.59) and mental health-related quality of life (r_ps_ = −0.54). Furthermore, a strong correlation was found between the total score on the self-report questionnaire and domain mental health-related quality of life (*r* = 0.46).

### Degree of agreement between professionals and adolescents

The degree of agreement between professionals and adolescents varied from no agreement to fair agreement (Table 6). The degree of agreement, in accordance with Altman’s guidelines [34], was fair in four domains: finances (*k* = 0.22), housing (*k* = 0.28), domestic relations (*k* = 0.21), and judicial (*k* = 0.21). The degree of agreement was poor for the domains Day-time activities (*k* = 0.07), mental health (*k* = 0.15), physical health (*k* = 0.17), and addiction (*k =* 0.18). No agreement (all *p*s > 0.05) was found in the three remaining domains (activities daily life, social network, and community participation).

**Table 6:** Degree of agreement between professionals’ and adolescents’ ratings of self-sufficiency (*n* = 224)

Self-sufficiency	Weighted kappa
Finances	0.22
Day-time activities	0.07
Housing	0.28
Domestic relations	0.21
Mental health	0.15
Physical health	0.17
Addiction	0.18
Activities daily life	0.004^a^
Social network	0.01^a^
Community participation	−0.003^a^
Judicial	0.21

^a^Non-significant correlations; all other correlations were significant at *p* <0.01

## Discussion

Both the self-report questionnaire assessing self-sufficiency and the SSM-D applied in this study seem to possess adequate psychometric properties in a group of vulnerable adolescents. The internal consistency was satisfactory. For most of the domains, there was also poor or fair agreement between professionals and adolescents.

More specifically, various minor to strong correlations were found between the domains of both the self-report questionnaire assessing self-sufficiency and SSM-D, on the one hand, and the related constructs, on the other. This is in line with our hypothesis as conceptual differences existed between the domains and the related constructs that were measured. Furthermore, different raters were used and this is reflected by the higher correlations that were found between the domains of the self-report questionnaire assessing self-sufficiency and the adolescent-reported related constructs than between the domains of the SSM-D and the adolescent-reported related constructs.

A low degree of agreement between informants is in line with previous research. For example, a small mean correlation (*r* = 0.22) between subjects and other informants was found when using questionnaires to measure adolescents’ psychopathology [18]. Furthermore, ‘needs’, as measured with an assessment instrument using areas of life related to the self-sufficiency domains (i.e. Camberwell Assessment of Need), are often assessed differently by professionals and clients [20].

There are several factors that could have decreased the degree of agreement between adolescents and professionals. First, the self-report questionnaire has, as opposed to the SSM-D, no indicators that specify each level of self-sufficiency, which may have contributed to a lower degree of agreement. A second potential explanation is that the subjective norms of professionals and adolescents differ from each other [19]. For example, professionals may have judged an adolescent as not being self-sufficient in the “day-time activities” domain because the adolescent in question had been truanting during the past week, while the adolescent him/herself might not see this as a problem if the truanting only happens occasionally. Third, it could be that adolescents are only partially aware of their problems [21], such as having “bad” friends or being addicted, whereas professionals may be able to assess these problems better. Fourth, professionals cannot observe all aspects of the life of an adolescent and they depend on what the adolescent tells them [19].

The low degree of agreement between adolescents and professionals indicates that both informants can provide different information on the self-sufficiency of adolescents [18]. Since there is no golden standard against which to validate measures of adolescent functioning in the various domains, it is essential to use the contributions of different informants to get a more complete picture of the problems adolescents are dealing with [18]. Having the adolescent complete the self-report questionnaire assessing self-sufficiency prior to the consultation with the professional could encourage professionals to also pay attention to adolescents’ views on their self-sufficiency and help them to determine which crucial aspect (s) to focus their discussions on [35, 36]. Furthermore, previous research has shown that completing a questionnaire on topics that are relevant to the consultation familiarises the adolescent with the topics the professional will bring up and better enables the adolescent to actively participate in the consultation [37].

Nevertheless, we recommend further improvement of the self-report questionnaire assessing self-sufficiency with respect to user-friendliness. In contrast to the SSM-D, which was completed by professionals, the self-report questionnaire has no indicators that specify each level of self-sufficiency (from 1 to 5) per domain yet, but only gives a short description of the content of the domain. In order to make the self-report questionnaire more user-friendly and each level of self-sufficiency easier to interpret, it is desirable to add indicators that specify each level of self-sufficiency per domain. Preferably, these indicators should correspond with the indicators available for professionals.

A strength of the study is the high response rate among a vulnerable population. A high percentage of the adolescents in our sample suffer from depressive symptoms and often engage in behaviours that negatively impact their health, such as substance abuse [23]. Given the fact that the level of self-sufficiency in the different domains was relatively high in this study, the results seem to suggest that adolescents in vocational education are often – still – able to deal with problems they encounter in daily life. Furthermore, about 11 % of the adolescents were already parents. Recently, additional self-sufficiency domains on parenting have been developed. Since about 11 % of the adolescents were already parents, it would be of interest to include these domains in future research on this population. However, the present study also has its limitations. The study relied on adolescents in vocational education. Therefore, the psychometric properties of both the self-report questionnaire assessing self-sufficiency and the SSM-D remain to be established in other settings and populations. Furthermore, the temporal stability of both instruments could not be examined. The concurrent validity could also not be examined for three domains (i.e. domestic relations, activities daily life, and social network) because no related constructs were measured. Moreover, conceptual differences existed between the other self-sufficiency domains and related constructs. A comparison of ratings for the self-sufficiency domains with ratings for an instrument that is as closely related to the domains as possible (e.g. Camberwell Assessment of Need) [20] would have strengthened this study.

## Conclusion

In conclusion, the results of this study show that both the self-report questionnaire assessing self-sufficiency and the SSM-D seem to possess adequate psychometric properties. Future research is necessary to investigate whether the results presented here can be replicated in different settings and populations as well as to investigate additional psychometric properties. We recommend using the adolescent-report questionnaire assessing self-sufficiency and the SSM-D concurrently to get a more complete picture of adolescent self-sufficiency. Both instruments express functioning in terms of levels of self-sufficiency in several domains, and can be considered for screening, monitoring or evaluation purposes. The instruments can be used during consultations with a professional to determine the functional strengths and areas for improvement. Furthermore, both instruments can be used to increase transparency in the decision-making processes in healthcare systems [38]. A great advantage of the self-report questionnaire assessing self-sufficiency and the SSM-D is that both versions can be completed in a short time, are freely available and can be used in a group of vulnerable adolescents.

## Additional file

Additional file 1:
**Polyserial correlations between each domain of self-sufficiency and the total score on SSM-D, and between each domain and the total score on the self-report questionnaire, were assessed.** (DOCX 12 kb)

## Abbreviations

SSM: Self-sufficiency matrix
SSM-D: Dutch version of the Self-sufficiency matrix
NTR: Netherlands trial register
MHI-5: Mental health inventory
CES-D: Center for epidemiologic studies depression scale
SF-12: Short form-12 health survey
r^rb^: Rank biserial correlation
r^ps^: Polyserial correlation
r^pc^: Polychoric correlation
r: Pearson correlation
k: kappa
