# Supplementary material for: Psychometric properties of self-sufficiency assessment tools in adolescents in vocational education
Source: BMC Psychol. 2015 Sep 25;3:33. doi: 10.1186/s40359-015-0091-2 (PMC4583751; doi:10.1186/s40359-015-0091-2)
Supplement: Additional file 1: — Polyserial correlations between each domain of self-sufficiency and the total score on SSM-D, and between each domain and the total score on the self-report questionnaire, were assessed. (DOCX 12 kb) [file 40359_2015_91_MOESM1_ESM.docx]

**Additional file 1.** (Polyserial) correlations between (professionals’ and adolescents’ ratings on) each domain and the total score

|  | **Total score on SSM-D** | **Total score on self-report** |
| --- | --- | --- |
| Finances | 0.64 | 0.64 |
| Day-time activities | 0.57 | 0.63 |
| Housing | 0.71 | 0.66 |
| Domestic relations | 0.71 | 0.67 |
| Mental health | 0.60 | 0.74 |
| Physical health | 0.28 | 0.60 |
| Addiction | 0.43 | 0.57 |
| Activities daily life | 0.28 | 0.55 |
| Social network | 0.76 | 0.68 |
| Community participation | 0.60 | 0.63 |
| Judicial | 0.57 | 0.58 |

*Note:* all correlations were significant at *p* <0.001.
